# Combination of Epigallocatechin Gallate and Sulforaphane Counteracts In Vitro Oxidative Stress and Delays Stemness Loss of Amniotic Fluid Stem Cells

**DOI:** 10.1155/2018/5263985

**Published:** 2018-12-17

**Authors:** Pasquale Marrazzo, Cristina Angeloni, Michela Freschi, Antonello Lorenzini, Cecilia Prata, Tullia Maraldi, Silvana Hrelia

**Affiliations:** ^1^Department for Life Quality Studies, Alma Mater Studiorum, University of Bologna, Corso d'Augusto 237, 47921 Rimini, Italy; ^2^School of Pharmacy, University of Camerino, Via Gentile III da Varano, 62032 Camerino, Italy; ^3^Department of Biomedical and Neuromotor Sciences, University of Bologna, Via Irnerio 48, 40126 Bologna, Italy; ^4^Department of Pharmacy and Biotechnology, Alma Mater Studiorum, University of Bologna, Via Irnerio 48, 40126 Bologna, Italy; ^5^Department of Surgery, Medicine, Dentistry and Morphological Sciences, University of Modena and Reggio Emilia, Policlinico, Via del Pozzo 71, 41124 Modena, Italy

## Abstract

Amniotic fluid stem cells (AFSCs) are characterized *in vivo* by a unique niche guarantying their homeostatic role in the body. Maintaining the functionality of stem cells *ex vivo* for clinical applications requires a continuous improvement of cell culture conditions. Cellular redox status plays an important role in stem cell biology as long as reactive oxygen species (ROS) concentration is finely regulated and their adverse effects are excluded. The aim of this study was to investigate the protective effect of two antioxidants, sulforaphane (SF) and epigallocatechin gallate (EGCG), against *in vitro* oxidative stress due to hyperoxia and freeze-thawing cycles in AFSCs. Human AFSCs were isolated and characterized from healthy subjects. Assays of metabolic function and antioxidant activity were performed to investigate the effect of SF and EGCG cotreatment on AFSCs. Real-time PCR was used to investigate the effect of the cotreatment on pluripotency, senescence, osteogenic and adipogenic markers, and antioxidant enzymes. Alkaline phosphatase assays and Alizarin Red staining were used to confirm osteogenic differentiation. The cotreatment with SF and EGCG was effective in reducing ROS production, increasing GSH levels, and enhancing the endogenous antioxidant defences through the upregulation of glutathione reductase, NAD(P)H:quinone oxidoreductase-1, and thioredoxin reductase. Intriguingly, the cotreatment sustained the stemness state by upregulating pluripotency markers such as OCT4 and NANOG. Moreover, the cotreatment influenced senescence-associated gene markers in respect to untreated cells. The cotreatment upregulated osteogenic gene markers and promoted osteogenic differentiation *in vitro*. SF and EGCG can be used in combination in AFSC culture as a strategy to preserve stem cell functionality.

## 1. Introduction

Amniotic fluid represents a promising source of cells and is free of ethical issues for regenerative medicine, including cell-based [1] and cell-free [2, 3] therapy. Human amniotic fluid stem cells (AFSCs) resemble different features from both embryonic stem cells (ESCs) and mesenchymal stromal/stem cells (MSCs). AFSCs have a valuable stemness profile since they express ESC-associated pluripotency markers such as OCT4 [4] NANOG [5], and SOX2 [6]. On the other hand, they share with MSCs with the absence of tumorigenicity and display a great immunomodulatory activity [7]. A substantial obstacle in producing clinical grade stem cells is the need for GMP culture conditions that must be xenofree during cryopreservation and postthawing of the cells. Unfortunately, AFSCs, in comparison to other MSC sources, are isolated in a relatively low number and need to be expanded prior and after their banking for clinical trials [6]. Moreover, freshly isolated stem cells are exposed to artificial physicochemical environments, quite different from those present *in vivo*, that can lead to a loose of the original functionality of the cells. Indeed, potential stem cell self-renewal and differentiation are critically regulated *in vivo* by oxygen and ROS concentrations that characterize their niche. Low levels of ROS are involved in physiological processes as proliferation and lineage specification; meanwhile, excessive levels of oxygen cause them a detrimental oxidative stress. *In vitro* cell cultures experience an atmospheric oxygen tension that is much higher than that found in tissues like bone marrow [8], umbilical cord blood [9], liver, and lung [10]. Oxygen is barely measurable in amniotic liquid [11]. Particularly, stem cells *in vivo* are located in niches where oxygen tension is extremely low (1-4%) [12] and hypoxic environments support the undifferentiated state of the stem cell [13, 14]. Although the organisms possess complex antioxidant systems to counteract ROS deleterious effects, it is unlikely that they are able to face the abnormal oxygen tension observed *in vitro*. Moreover, cryopreservation and thawing further increase ROS levels exacerbating oxidative stress *in vitro* [15]. To harness the robust therapeutic potential of AFSCs, a consistent and economical method to fight the deleterious effect of ROS induced by *in vitro* environment is essential. In this context, natural dietary compounds with antioxidant activity are optimal candidates to be included in stem cell culture protocols because of their safety and their ability to control oxidative stress. ARE-gene battery activated by Nrf2, the major stress response regulator evolved by mammalian cells [16], has been demonstrated to be induced by sulforaphane (SF) in different cell types [17, 18] and by epigallocatechin gallate (EGCG) [19] in MSCs, too [20]. In this study, we evaluated the effect of a combined supplementation with SF and EGCG on replicative capacity, redox state, senescence, and stemness of human AFSCs.

## 2. Materials and Methods

### 2.1. Materials

The materials used include alpha-modified eagle medium (*α*MEM), L-glutamine, penicillin/streptomycin, Accutase, 3-(4,5-dimethylthiazol-2-yl)-2,5-diphenyl-tetrazolium bromide (MTT), dimethyl sulfoxide (DMSO), EGCG, 2′-7′-dichlorodihydrofluorescein diacetate (DCFH-DA), monochlorobimane (MCB), paraformaldehyde 4% (PF 4%), 5-bromo-4-chloro-3-indolyl phosphate-Nitro Blue Tetrazolium (BCIP/NBT®) Liquid Substrate System, Alizarin Red staining, Senescence Cells Histochemical Staining Kit, protease inhibitor cocktail, rabbit anti-*β*-actin, primers listed in Table 1 (Sigma Chemical, St. Louis, USA), D,L-sulforaphane, SF (LKT Laboratories, Minneapolis, USA), fetal bovine serum (FBS) (EuroClone), Prestoblue®, StemPro® Osteogenesis Differentiation Kit (Thermo Scientific, Waltham, USA), Alkaline Phosphatase Assay Kit (Abcam, Cambridge, UK), RNeasy Mini kit (Qiagen, Germantown, USA), iScript cDNA Synthesis Kit, SsoAdvanced Universal SYBR Green Supermix Kit (Bio-Rad, Hercules, USA), Supersignal substrate chemiluminescence detection kit (Pierce, Rockford, USA), Immobilon-P membranes (Millipore, Waltham, USA), rabbit anti-p16 (Abcam, Cambridge, UK), and mouse anti-pH2A (Millipore, CA, USA).

All other chemicals of the highest analytical grade were purchased from Sigma Chemical, unless otherwise stated.

### 2.2. Cell Isolation and Culture

Human amniotic fluid was collected and processed as previously reported [21]. Human AFSCs were isolated as previously reported [4]. Briefly, amniocentesis cultures were harvested by trypsinization and subjected to c-Kit immunoselection by MACS technology (Miltenyi Biotec). Growth culture media are *α*MEM supplemented with 20% of FBS, 2 mM L-glutamine, 100 U/ml penicillin, and 100 *μ*g/ml streptomycin. Cells were passaged once or twice in a week, not subcultured above 1 : 3 ratio.

During the preamniocentesis interview, pregnant women were informed about the purpose of the study and any related risks. The informed consents were obtained, in accordance with the Italian law and the guidelines of the ethics committee (protocol 2015/0004362 of 02.24.2015). Informed consent, as well as all documentation relating to the invasive procedure, was signed by the pregnant women and by a specialist before continuing the exam.

### 2.3. MTT Assay

AFSCs were seeded in 96-well plates at a density of 10000 cells/well (day 0) in 200 *μ*l of a culture medium, 4 replicates for each condition. At the end of each experiment, 0.5 mg/ml MTT was added and incubated for 1.5 h at 37°C. After incubation, MTT solution was removed and DMSO was added to solubilize the formazan salts. The absorbance was measured at *λ* = 595 nm using a microplate spectrophotometer (VICTOR3 V Multilabel Counter; PerkinElmer, Wellesley, MA, USA).

### 2.4. ROS Detection

To evaluate intracellular ROS levels, dichlorodihydrofluorescein diacetate (DCFH-DA) assay was performed as previously described [22]. Cells were seeded in a 96-well plate at density of 10000 cells/cm^2^, 4 replicates for each condition, treated with EGCG and SF for 72 hours soon after thawing, otherwise chronically during expansions. Cell culture medium was removed, and the 5 *μ*M DCFH-DA was incubated in *α*MEM 1% FBS without phenol red for 30 min, at 37°C and 5% CO_2_. The cell culture plate was washed with PBS, and fluorescence of the cells was read at 485 nm (excitation) and 535 nm (emission) using the VICTOR multilabel plate reader (PerkinElmer).

### 2.5. PrestoBlue® Assay

Cell viability PrestoBlue® reagent is a ready-to-use resazurin-based solution that functions as a cell health indicator by using the reducing power of living cells. PrestoBlue® reagent was prepared in growth culture media without phenol red. AFSCs were seeded at 10000 cells/well in 96-well plates (day 0) in 200 *μ*l of a culture medium, 4 replicates for each condition. At 24 h and 72 h, the cell culture medium was replaced with 100 *μ*l of PrestoBlue working solution and incubated at 37°C and 5% CO_2_. After 2 hours, the incubated PrestoBlue volumes were collected in a new 96-well plate and the cells were refeed with a fresh culture medium. The absorbance was read at *λ* = 570 nm (experimental) and *λ* = 600 nm (reference wavelength for normalization) using the VICTOR multilabel plate reader (PerkinElmer).

### 2.6. Population Doublings

AFSCs were subcultured until 75% of confluence. Cells beyond confluence were detached using Accutase solution. Cell suspension aliquots were stained in trypan blue and counted by Countess system (Thermo Fisher, Waltham, USA). Diluted cell suspension was seeded again in T25 flasks. To calculate cumulative population doubling (cPD), the following formula was applied to all samples for each experimental group:

(1) $$\begin{array}{c} PD=\frac{\log_{10}NH-\log_{10}NS}{\log_{10}2}, \end{array}$$

where PD is population doubling, NS is the cell number at seeding, and NH is the cell number at harvest. To calculate the cumulative number of population doublings (cPDs), the PDs determined for each passage were summed.

### 2.7. Glutathione Detection

To evaluate reduced GSH levels, monochlorobimane (MCB) assay was performed as previously reported [23]. AFSCs were seeded in a 96-well plate at a density of 15000 cells/cm^2^, 4 replicates for each condition, treated for 72 h soon after thawing, otherwise chronically during expansion. Cell culture medium was removed, and 50 *μ*M MCB was incubated in *α*MEM without phenol red supplemented with 1% FBS for 30 min, at 37°C and 5% CO_2_. The cells were washed in PBS, and fluorescence of the cells was measured at 355 nm (excitation) and 460 nm (emission) using the VICTOR multilabel plate reader (PerkinElmer). Cellular autofluorescence was subtracted as a background using the values of the wells not incubated with the probe.

### 2.8. SA-*β*-galactosidase Assay

Expression of senescence-associated *β*-galactosidase (SA-*β*-gal) in AFSCs was analysed using Senescence Cells Histochemical Staining Kit, according to the manufacturer's instructions. Increased *β*-galactosidase expression detected by blue coloured staining has been reported to increase in senescent cells [24]. Briefly, subconfluent AFSCs were washed in PSB and fixed for 4 min. Staining solution was incubated at 37°C overnight. SA-*β*-gal-positive stained cells were examined under the light microscope with 10x magnification. Representative pictures from random fields were acquired by a colour camera. For quantitative analysis, at least 150 total cells from each condition were counted across multiple fields. Percentages of senescent cells vs. total cells were obtained based on the counts by two operators.

### 2.9. *In Vitro* Osteogenesis

Osteogenesis induction was performed with StemPro® Osteogenesis Differentiation Kit according to the manufacturer's instructions. Briefly, AFSCs were plated on various substrates and cultured up to 2 days before switching to differentiation medium. The cells were subsequently cultured for 14 days replacing the medium twice a week.

For alkaline phosphatase detection, after cell culture medium removal, cells were washed in PBS and fixed for 10 min in PF 4%. Cells were then washed in H_2_O; BCIP®/NBT Liquid Substrate System was added, and cells were incubated overnight at room temperature. Alkaline phosphatase converts BCIP to a product that reduces NBT to a blue-purple precipitate. Samples were finally washed in H_2_O.

For Alizarin Red staining, cells were washed in PBS and fixed for 10 min in PF 4%. Cells were then washed in H_2_O, and 2% Alizarin Red solution was added for 30 min at room temperature. Red staining is indicative of calcium deposits. Samples were finally washed in H_2_O. To quantify the Alizarin Red S staining, samples were washed with PBS and then 10% cetylpyridinium chloride was added and incubated for 20 min to elute the stain. 10 *μ*l of this elution was added to 90 *μ*l of water and read at 485 nm using a spectrophotometer (VICTOR multilabel plate reader, PerkinElmer) [25]. To quantify the alkaline phosphatase, Alkaline Phosphatase Assay Kit was used following the manufacturer's instruction, measuring absorbance at 390 nm using a spectrophotometer (VICTOR multilabel plate reader, PerkinElmer).

### 2.10. Real-Time PCR

To evaluate the expression of antioxidant enzymes, the cells were seeded in 6-well plates at 70% confluence and treated with SF and EGCG and, after 6 hours, RNA was extracted. To evaluate the levels of stemness and senescence, the cells were gently thawed in complete growth media and pelleted and split in treated and not treated samples. After 72 hours, RNA was extracted; alternatively, the samples were expanded continuing to receive the treatment or not. RNA was isolated using RNeasy Mini kit following the manufacturer's protocol. Starting from 1 *μ*g of the extracted RNA, the cDNA was obtained using iScript cDNA Synthesis Kit following the manufacturer's protocol. Real-time PCR was performed using SsoAdvanced Universal SYBR Green Supermix following the manufacturer's protocol (temperature). Real-time PCR reaction was carried out in a total volume of 10 *μ*l loading 250 ng of cDNA and 500 nM of each primer. The cDNA amplification was performed by activating the polymerase for 30 s at 95°C, followed by 40 cycles of 5 s at 95°C and 30 s at 60°C. Primer sequences used in this study are listed in Table 1. Normalized expression levels were calculated relative to control cells according to the 2^−ΔΔCT^ method.

### 2.11. Immunofluorescence and Confocal Microscopy

For immunofluorescence analysis, samples were processed as previously described [6]. Confocal imaging was performed by a Nikon A1 confocal laser scanning microscope. Primary antibodies were raised against the following molecules: mouse-pH2A and Rabbit-p16. The confocal serial sections were processed with ImageJ software to obtain three-dimensional projections. The image rendering was performed by Adobe Photoshop software.

### 2.12. Western Blotting

Cell extracts were obtained as described by Beretti et al. [21]. Briefly, subconfluent cells were extracted by the addition of AT lysis buffer (20 mM Tris-Cl, pH 7.0; 1% Nonidet P-40; 150 mM NaCl; 10% glycerol; 10 mM EDTA; 20 mM NaF; 5 mM sodium pyrophosphate; and 1 mM Na_3_VO_4_) and freshly added protease inhibitor cocktail at 4°C for 30 min. Lysates were sonicated, cleared by centrifugation, immediately boiled in SDS sample buffer, and centrifuged. Supernatants were loaded onto SDS-polyacrylamide gel, blotted on Immobilon-P membranes, and processed by western blot with the indicated antibodies, detected by SuperSignal substrate chemiluminescence detection kit. Quantitation of the signal was obtained by chemiluminescence detection on a Kodak Image Station 440CF and analysis with the Kodak 1D Image software. Primary antibodies were raised against the following molecules: rabbit-p16 and rabbit-*β*-actin.

### 2.13. Statistics

Statistical analysis and plot layout were obtained by using GraphPad Prism® release 6.0 software. Statistics performed are described in figure legends.

## 3. Results

### 3.1. EGCG and SF Effect on Cell Viability

**Figure 1:**
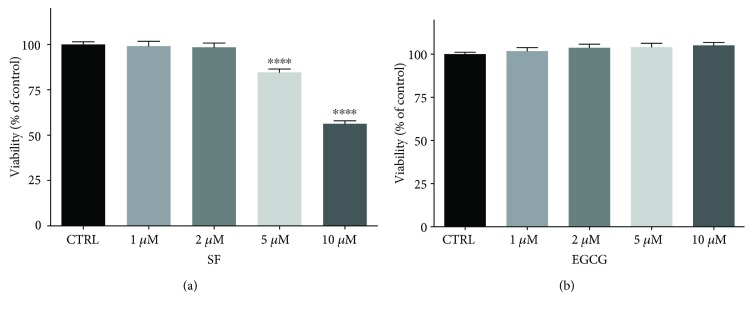
Viability of AFSCs treated with SF and EGCG. Cells were treated for 3 days with increasing concentrations of (a) SF or (b) EGCG. Viability was measured by MTT assay, as reported in Materials and Methods. Each bar represents means ± SEM of 3 independent experiments. Data were analysed by one-way ANOVA followed by Bonferroni's test. ^∗∗∗∗^ *p* < 0.0001 with respect to CTRL.

SF or EGCG was supplemented in the culture medium at concentrations ranging from 1 to 10 *μ*M. After 3 days, cytotoxicity was evaluated. Viability of cells treated with 1 *μ*M and 2 *μ*M SF was comparable to untreated control cells, while higher concentrations significantly reduced AFSC viability (Figure 1(a)). EGCG treatment did not influence cell viability at any tested concentrations (Figure 1(b)).

### 3.2. SF and EGCG Treatments Reduce the Intracellular ROS Level

**Figure 2:**
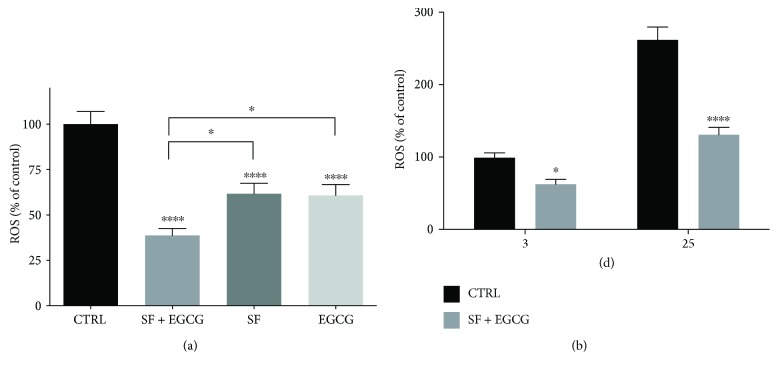
Effect of AFSC treatment with SF and EGCG on the ROS intracellular level. (a) Cells were treated or cotreated for 3 days with 1 *μ*M SF and 10 *μ*M EGCG. ROS levels were evaluated by DCFH-DA fluorometric assay, as reported in Materials and Methods. Each bar represents means ± SEM of 3 independent experiments. Data were analysed by one-way ANOVA followed by Bonferroni's test. ^∗^ *p* < 0.05, ^∗∗∗∗^ *p* < 0.0001 with respect to relative control. (b) Cells were cotreated for 3 days after their thawing or (c) chronically treated for 25 days. The ROS level was evaluated by DCFH-DA fluorometric assay as reported in Materials and Methods. Each bar represents means ± SEM of 3 independent experiments. Data were analysed by one-way ANOVA followed by Bonferroni's test. ^∗^ *p* < 0.05, ^∗∗∗∗^p < 0.0001 with respect to relative control.

As ROS are able to impair stem cell functionality [15], we verified the effect of SF and EGCG treatment on intracellular ROS level. Cells were treated with non-toxic concentrations of SF or EGCG before DCFH-DA assay (Supplementary Materials, Figure S1). One and 2 *μ*M SF significantly reduced the ROS level; meanwhile, only 10 *μ*M EGCG was effective in reducing basal ROS levels. On the basis of these results, to evaluate the potential additive effect of the combined treatment with SF and EGCG, we selected 1 *μ*M SF and 10 *μ*M EGCG for the cotreatment (Figure 2(a)). Noteworthy, EGCG-SF cotreatment for 3 days was able to significantly reduce the ROS level in respect to cells treated with SF and EGCG alone. As the combination of the two natural compounds was more effective than the treatment with the single compounds, only the cotreatment was utilized for the subsequent experiments. As thawing is a critical event for stem cells due to hyperoxia and subsequent ROS production [15], cells were cotreated soon after thawing with SF and EGCG for 3 days before ROS evaluation (Figure 2(b)). As we observed an increase in the ROS level during cell passages, we evaluated the effect of a chronic treatment. AFSCs were exposed to the cotreatment starting from thawing until 25 d of culture (Figure 2(b)). In agreement with the acute treatment, the long-term treatment significantly reduced the intracellular ROS level.

### 3.3. EGCG and SF Cotreatment Does Not Affect Expansion of AFSCs

**Figure 3:**
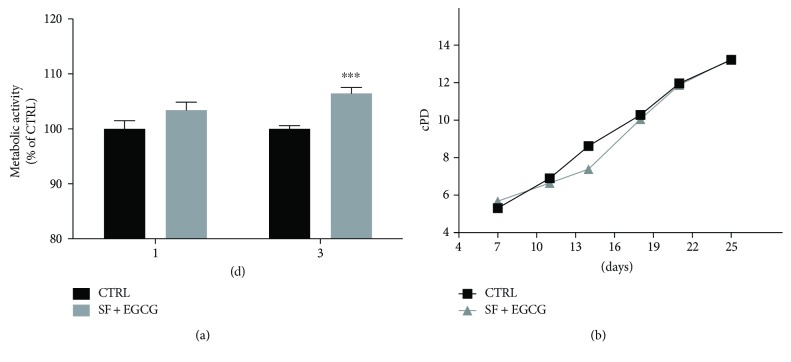
Effect of the cotreatment with SF and EGCG on AFSC viability and proliferation. (a) Cells were cotreated with SF and EGCG and metabolic activity evaluated by PrestoBlue assay, as reported in Materials and Methods at 1 day and 3 days after treatment. Each bar represents means ± SEM of 3 independent experiments. Data were analysed by a Mann–Whitney test. ^∗∗∗^ *p* < 0.001 with respect to control cells. (b) Cells were cotreated chronically and expanded for 25 days in culture, and cPD was calculated as reported in Materials and Methods. Each dot represents means of 3 independent experiments.

Cells were cotreated with EGCG and SF, and the metabolic activity was evaluated by PrestoBlue assay. Interestingly, EGCG-SF treatment significantly increased the metabolic rate in respect to control cells at 3 days (Figure 3(a)). Cells chronically cotreated with EGCG and SF were compared to untreated cells for population doublings (PD) obtained by semiautomatic cell counting. The chronic cotreatment of AFSCs with the antioxidants did not increase cell numbers during AFSC expansion; indeed, PDs from treated and not treated cells were comparable at each time point (Figure 3(b)).

### 3.4. EGCG and SF Cotreatment Enhances the Antioxidant Defence System

**Figure 4:**
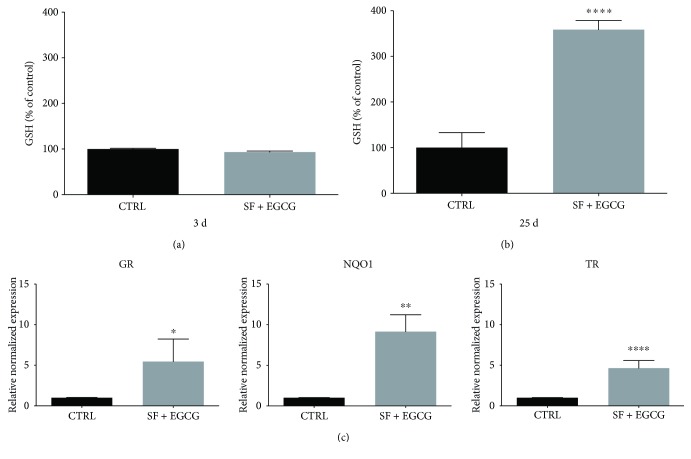
Effect of the cotreatment with SF and EGCG on AFSC antioxidant defences. (a) Cells were cotreated for 3 days after thawing or (b) chronically treated for 25 days. GSH levels were evaluated by MCB fluorimetric assay as reported in Materials and Methods. Each bar represents means ± SEM of 3 independent experiments. Data were analysed by a Mann–Whitney test. ^∗∗∗∗^ *p* < 0.0001 with respect to the control. (c) Effect of the treatment with SF and EGCG on the expression of GR, TR, and NQO1 in AFSCs. Cells were cotreated for 6 h after thawing. Total RNA was isolated, and the mRNA, as expression of target genes, was quantified using RT-PCR normalized to housekeeping gene as reported in Materials and Methods. Triplicate reactions were performed for each experiment. Each bar represents mean ± SEM of 3 independent experiments. Data were analysed by a Mann–Whitney test. ^∗^ *p* < 0.05, ^∗∗^ *p* < 0.01, and ^∗∗∗∗^ *p* < 0.0001.

In order to investigate the effect of the cotreatment on the endogenous antioxidant defence system, cells were exposed to 1 *μ*M SF and 10 *μ*M EGCG and GSH level and expression of GR, NQO1, and TR were evaluated (Figure 4). The GSH level were not influenced by a 3-day cotreatment (Figure 4(a)); nevertheless, it significantly increased after a chronic treatment for 25 days (Figure 4(b)). To evaluate antioxidant enzyme expression, cells were cotreated with SF and EGCG and then RNA was extracted and analysed by real-time PCR. Of note, the cotreatment was able to significantly upregulate GR, TR, and NQO1 (Figure 4(c)).

### 3.5. EGCG and SF Cotreatment Prolongs Stemness Markers and Delays the Expression of Senescence Markers

**Figure 5:**
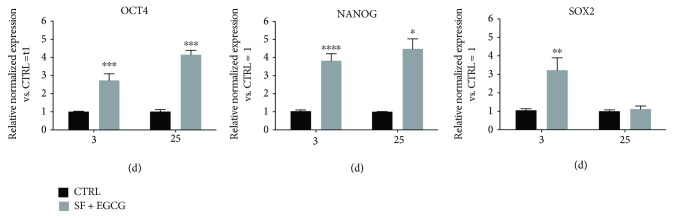
Effect of AFSC cotreatment with SF and EGCG on stemness. Total RNA was isolated, and mRNA expression of target genes was quantified using RT-PCR normalized to housekeeping genes as reported in Materials and Methods. Triplicate reactions were performed for each experiment. Cells were cotreated for 3 days after thawing or cotreated chronically during AFSC expansion for 25 days, and OCT4, NANOG, and SOX2 gene expression was analysed. Each bar represents the mean ± SEM of 3 independent experiments. Data were analysed by two-way ANOVA followed by Bonferroni's test. ^∗^ *p* < 0.05, ^∗∗^ *p* < 0.01, ^∗∗∗^ *p* < 0.001, and ^∗∗∗∗^ *p* < 0.0001.

**Figure 6:**
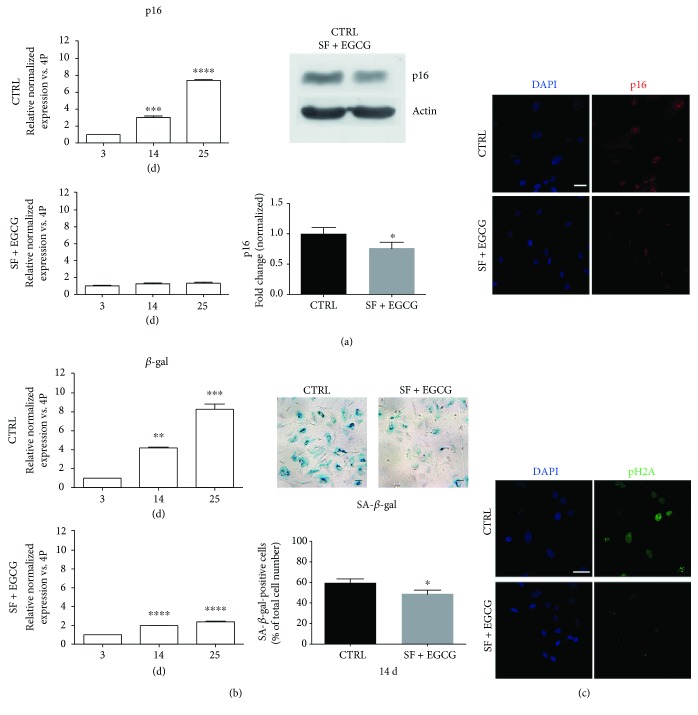
Effect of AFSC cotreatment with SF and EGCG on senescence-associated markers. (a) Cells were cotreated for 3 days (3 d), 14 days (14 d), and 25 days (25 d) and analysed for p16 gene expression by real-time PCR. Each bar represents mean ± SEM of 3 independent experiments. Data were analysed by one-way ANOVA followed by Bonferroni's test. ^∗∗^ *p* < 0.01, ^∗∗∗^ *p* < 0.001, and ^∗∗∗∗^ *p* < 0.0001; cells were cotreated for 14 days and analysed for p16 protein levels by western blot analysis. *β*-Actin detection was performed as a loading control. The graph shows densitometric analysis of western blot experiment. Data are representative of three independent experiments. Data were analysed by Student's test. ^∗^ *p* < 0.05. Representative confocal images of AFSC, cotreated or not for 14 d, double stained with DAPI (blue) and p16 (red). Scale bar = 10 *μ*M. (b) Cells were cotreated for 3 d, 14 d, and 25 d and analysed for *β*-gal gene expression by real-time PCR. Data were analysed by one-way ANOVA followed by Bonferroni's test. ^∗∗^ *p* < 0.01, ^∗∗∗^ *p* < 0.001, and ^∗∗∗∗^ *p* < 0.0001. Representative pictures of SA-*β*-gal staining and quantitative analysis of SA-*β*-gal-positive AFSCs are reported. Each bar represents means ± SEM of 3 independent experiments. Data were analysed by Student's test. ^∗^ *p* < 0.05. (c) Representative confocal images of AFSC, cotreated or not for 14 days, double stained with DAPI (blue) and pH2A (green). Scale bar = 10 *μ*M.

To study the effect of the cotreatment on stemness of AFSCs, gene expression of markers associated to pluripotency state, such as OCT4, NANOG, and SOX2 [26, 27], was evaluated by real-time PCR. AFSCs treated immediately after thawing for 3 days showed significantly higher levels of OCT4, NANOG, and SOX2 mRNAs, in comparison to untreated samples (Figure 5). The chronic treatment of cells during expansion influenced the expression of OCT4 and also NANOG. Indeed, cells at early and late passages showed a greater expression of these two markers in comparison to the untreated controls (Figure 5). Moreover, the cells expanded for consecutive passages were analysed by real-time PCR for the expression of senescence-associated markers (Figures 6(a) and 6(b)). The cells in the absence of treatment had a stronger increase in the expression of p16 and *β*-gal, as consequence of passaging, in comparison to the treated cells that showed only a limited increase (Figures 6(a) and 6(b)). Cotreatment effect on p16 has also been confirmed by western blot analysis and immunofluorescence (Figure 6(a)). SA-*β*-gal activity has been reduced by the cotreatment, as shown in Figure 6(b). The obtained results are consistent with the RT-PCR data. To strengthen these observations, we evaluated senescence-associated DNA damage foci (SDFs), a DNA damage response marker (Figure 6(c)). Of note, cotreated cells presented a lower number of nuclei with phospho-histone 2A (pH2A) foci than control cells, confirming the antisenescence effect of the cotreatment.

### 3.6. EGCG-SF Treatment Primes for Osteogenic Differentiation

**Figure 7:**
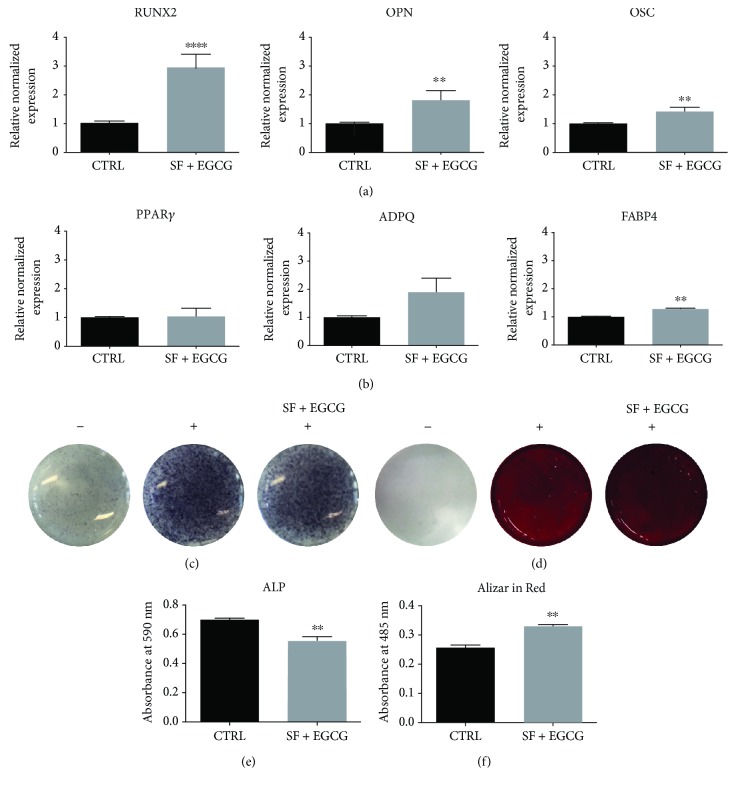
Effect of cotreatment with SF and EGCG on osteogenic differentiation potential. (a) Effect of the treatment with SF and EGCG after 9 days on the expression of RUNX2, OPN, and OSC osteogenic markers and (b) of the adipogenic markers PPAR*γ*, ADPQ, and FABP4 analysed by RT-PCR normalized to housekeeping genes as reported in Materials and Methods. Triplicate reactions were performed for each experiment. Each bar represents the mean ± SEM of 3 independent experiments. Data were analysed by a Mann–Whitney test. ^∗∗^ *p* < 0.01, ^∗∗∗∗^ *p* < 0.0001 in respect to the control (CTRL). (c) BCIP/NBT staining used to detect ALP activity in samples cotreated with SF and EGCG during osteogenic differentiation. (d) Alizarin Red staining used to detect calcium deposits in samples cotreated with SF and EGCG during osteogenic differentiation. (e) Spectrophotometric quantification of the results obtained with Alkaline Phosphatase Assay Kit. (f) Spectrophotometric quantification of solubilized Alizarin Red staining showed in (d). Data were analysed by unpaired *t*-test. ^∗∗^ *p* < 0.01, in respect to the control (CTRL). −: not induced to differentiation; +: induced to differentiation.

Since antioxidants can modulate differentiation of stem cells [28], AFSCs treated with EGCG and SF were analysed for lineage-specific master regulators of osteogenesis and adipogenesis (Figure 7). A 9-day treatment significantly upregulated the expression of osteogenic markers, such as RUNX2, osteopontin (OPN), and osteocalcin (OSC), unlike the expression of the adipogenic markers PPAR*γ* and adiponectin (ADPQ). Even if the cotreatment upregulated the adipogenic marker fatty acid-binding protein 4 (FABP4), this slight modulation could not be considered relevant from a biological point of view. To evaluate the effect of SF and EGCG cotreatment on *in vitro* osteogenic differentiation, osteogenic differentiation was induced supplementing the differentiation medium with EGCG and SF for 14 days. Interestingly, the cotreatment boosts up osteogenic differentiation as confirmed by alkaline phosphatase assays (Figures 7(c) and 7(e)) and Alizarin Red staining (Figures 7(d) and 7(f)).

## 4. Discussion

*In vivo* stem cells reside in a hypoxic niche that preserves MSC progenitor properties [29]; meanwhile, routinely, *in vitro* culture of stem cells is performed in atmospheric oxygen tension that leads to an abnormal production of ROS. Moreover, freeze-and-thaw cycles also contribute to oxidative stress [30]. The excess of intracellular ROS deeply impacts on stem cell functionality, e.g., postthawing recovery, proliferation, and differentiation ability [30]. To arm MSCs against these stresses is a challenge for basic and clinical research. Plant secondary metabolites carry out numerous interactions, and many phytochemicals show a potent antioxidant activity. Antioxidants may represent a tempting strategy in order to limit the oxidative stress *in vitro* of human stem cells. Nrf2-Keap1 is the most important stress response-related pathway [31], and among its activators, the flavonoid EGCG and the isothiocyanate SF have gained a great popularity and consideration mainly as chemopreventive [32, 33] and cardioprotective agents [34, 35].

On the basis of these premises, we explored the effect of a combined treatment with EGCG and SF on postthawing recovery and expansion of human AFSCs. These two phytochemicals were chosen since they possess different chemophysical properties (EGCG is more hydrophilic than SF) and because they could modulate various cell pathways. Many examples regarding the improving in protocols for cryobiology of stem cells are based on single antioxidant supplementation [36–38]. Our results show a higher efficacy of the cotreatment with SF and EGCG against oxidative stress in respect to the single antioxidant treatment. Importantly, the synergic effect of EGCG and SF on oxidative stress was observed after cell thawing, i.e., a time frame in which a burst of unavoidable ROS impairs the cells.

As it has been recently highlighted a positive correlation between GSH and stemness and general functionality in MSCs [39], we checked GSH levels that resulted a significant increase after a chronic cotreatment with the two antioxidants. The TRX and GSH systems are the major cellular antioxidant defence mechanisms, interacting with many cellular survival pathways [40]. Interestingly, EGCG and SF cotreatment upregulated both GR and TR, the antioxidant enzymes that preserve TRX and GSH homeostasis. Moreover, our data indicated an upregulation of another key antioxidant enzyme, NQO1, involved in the production of hydroquinone to counteract deleterious quinonic radical formation. Taken together, our data suggest that EGCG and SF counteract oxidative stress by enhancing the antioxidant defence system in AFSCs.

As highlighted above, cryopreservation is an artificial step that not rarely affects stem cell original features. In our experimental protocol, after cell thawing, the cotreatment with EGCG and SF induced the upregulation of the expression of pluripotency-associated markers such as OCT4, NANOG, and SOX2, in respect to control untreated cells. Thus, EGCG and SF may be proposed as “preservative” agents for the self-renewal of MSCs, retarding their spontaneous decrease of pluripotency with passaging overtime. Away from their *in vivo* niche, stem cells [41, 42] normally undergo senescence. In this context, ROS were known to contribute to senescence via specific pathways like the ones modulated by p16 and p38 [43, 44]. The expressions of the senescent markers were delayed in our experimental protocol. This antiaging effect is in agreement with the improved stemness marker profile we observed after a chronic exposure to EGCG and SF. These data suggest a reduced activation of ROS-induced senescence pathways [44]. Indeed, both EGCG [20] and SF [45] were shown to be involved in the repression of senescence *in vitro* in MSCs. Many biological functions of MSCs have been linked to microvesicles that act as paracrine factors. As microvesicles mirror the parental cell characteristics, delaying senescence could also preserve microvesicle efficacy [46].

Multipotent AFSCs can be committed to mesenchymal lineages, such as osteogenic and adipogenic lineage. Osteogenesis and adipogenesis in MSCs are phenotypes competing each other [47]. The pathways governing the osteogenic and adipogenic differentiation are modulated by two master regulators that are peroxisome proliferator-activated receptor-*γ* (PPAR*γ*) and runt-related transcription factor 2 (RUNX2). Inhibition of the adipogenesis specifically due to SF [48] and EGCG [49, 50] or generally due to Nrf2 activation [51] was reported. Interestingly, during cell expansion, chronic cotreatment counteracted OCT4 and NANOG physiological decrease; meanwhile, SOX2, a repressor of mesodermic differentiation [52], was comparable to control cells. Moreover, the addition of EGCG [53–55] and SF [56] was associated to a proosteogenic phenotype, so we investigated the effect of their cotreatment in osteogenic differentiation. A 9-day cotreatment promotes a proosteogenic profile in AFSCs, as evidenced by the upregulation of RUNX2 and other osteogenic markers like OSC and OPN. Thus, priming AFSCs with EGCG and SF may be a stimulation step to trigger osteogenic differentiation *in vitro*. Indeed, the expression of the adipogenesis marker PPAR*γ*, ADPQ, and FABP4 were not influenced by the cotreatment, suggesting that SF and EGCG do not affect adipogenic potential of AFSCs. Furthermore, samples were simultaneously cotreated and induced to differentiate for 14 days towards osteogenic lineage and were analysed by histochemistry. The cotreatment promoted the osteogenesis commitment, as seen by ALP histochemical staining and calcium deposition by Alizarin staining. Indeed, the cotreatment increases calcium deposition during osteogenic differentiation; meanwhile, ALP activity decreases, as expected, since during normal osteogenic differentiation, there is an initial peak in ALP production followed by a subsequent decrease as the cells mature and lay down mineral [57]. In this way, the proposed SF and EGCG cotreatment is at least compatible with osteogenic differentiation protocols, an important concern for the use of AFSCs in bone regenerative medicine and tissue engineering.

## 5. Conclusions

In summary, the number of cell passages, the maintenance of the self-renewal capacity, and the ability to counteract the external oxidative stress are critical key points to be considered for the quality control of AFSCs [58]. In our study, EGCG and SF were proposed as alternative bioderived additives for prolonging lifespan of functional *in vitro* AFSCs since a retained functionality is essential for enhancing the therapeutic potential of this promising stem cell population.

## Figures and Tables

**Table 1:** Primers used in RT-PCR. ^∗^Internal normalizer.

Gene	Sequence	RefSeq accession no.
RPS18^∗^	Fw CAGAAGGATGTAAAGGATGG	**NM_022551**
	Rv TATTTCTTCTTGGACACACC	
GAPDH^∗^	Fw ACAGTTGCCATGTAGACC	**NM_002046**
	Rv TTGAGCACAGGGTACTTTA	
NQO1	Fw AGTATCCACAATAGCTGACG	**NM_000903**
	Rv TTTGTGGGTCTGTAGAAATG	
GR	Fw GACCTATTCAACGAGCTTTAC	**NM_000637**
	Rv CAACCACCTTTTCTTCCTTG	
TR	Fw AGACAGTTAAGCATGATTGG	**NM_001093771**
	Rv AATTGCCCATAAGCATTCTC	
NANOG	Fw CCAGAACCAGAGAATGAAATC	**NM_024865**
	Rv TGGTGGTAGGAAGAGTAAAG	
SOX2	Fw ATAATAACAATCATCGGCGG	**NM_003106**
	Rv AAAAAGAGAGAGGCAAACTG	
OCT4	Fw AGAGAAAGCGAACCAGTATC	**NM_002701.5**
	Rv TTACAGAACCACACTCGG	
*β*-gal	Fw GACAGTACCAGTTTTCTGAG	**NM_000404**
	Rv ATAGACTCTTTCTCTAGCAGC	
p16	Fw AGCATGGAGCCTTCG	**NM_000077**
	Rv ATCATGACCTGGATCGG	
RUNX2	Fw GCAGTATTTACAAGAGGG	**NM_001015051**
	Rv TCCCAAAAGAAGTTTTGCTG	
OPN	Fw CATCTCAGAAGCAGAATCTC	**NM_001251830**
	Rv GAAGGGTCTCTTGTTTAAAGTC	
OSC	Fw TTCTTTCCTCTTCCCCTTG	**NM_199173**
	Rv CCTCTTCTGGAGTTTATTTGG	
PPAR*γ*	Fw AAAGAAGCCAACACTAAACC	**NM_138712**
	Rv TGGTCATTTCGTTAAAGGC	
ADPQ	Fw GGTCTTATTGGTCCTAAGGG	NM_ 001177800
	Rv GTAGAAGATCTTGGTAAAGCG	
FABP4	Fw CAAGAGCACCATAACCTTAG	NM_ 001442
	Rv CTCGTTTTCTCTTTATGGTGG	

## Data Availability

All data generated and/or analysed during this study are available from the corresponding author on reasonable request.

## Abbreviations

ADPQ: Adiponectin
AFSCs: Amniotic fluid stem cells
ALP: Alkaline phosphatase
BCIP: Bromo-4-chloro-3-indolyl phosphate
cPD: Population doubling
DCFH-DA: 2′-7′-Dichlorodihydrofluorescein diacetate
DMSO: Dimethyl sulfoxide
EGCG: Epigallocatechin gallate
FABP4: Fatty acid-binding protein 4
FBS: Fetal bovine serum
GR: Glutathione reductase
GSH: Glutathione
MCB: Monochlorobimane
MTT: 3-(4,5-Dimethylthiazol-2-yl)-2,5-diphenyl-tetrazolium bromide
NBT: Nitro blue tetrazolium
NANOG: Nanog homeobox
NQO1: NAD(P)H:quinone oxidoreductase-1
OCT4: Octamer-binding transcription factor 4
OPN: Osteopontin
OSC: Osteocalcin
PBS: Phosphate-buffered saline
PF: Paraformaldehyde
pH2A: Phospho-histone 2A
PPAR*γ*: Peroxisome proliferator-activated receptor-*γ*
ROS: Reactive oxygen species
RUNX2: Runt-related transcription factor 2
SF: Sulforaphane
SOX2: SRY-box containing protein 2
TR: Thioredoxin reductase
TRX: Thioredoxin
*α*MEM: Alpha-modified eagle medium
*β*-gal: *β*-Galactosidase.

## Supplementary Materials

Supplementary Materials Figure S1: effect of the treatment with SF and EGCG on the ROS intracellular level.
