# Supplementary material for: Combination of Epigallocatechin Gallate and Sulforaphane Counteracts In Vitro Oxidative Stress and Delays Stemness Loss of Amniotic Fluid Stem Cells
Source: Oxid Med Cell Longev. 2018 Dec 17;2018:5263985. doi: 10.1155/2018/5263985 (PMC6311758; doi:10.1155/2018/5263985)
Supplement: Supplementary Materials — Figure S1: effect of the treatment with SF and EGCG on the ROS intracellular level. [file 5263985.f1.docx]

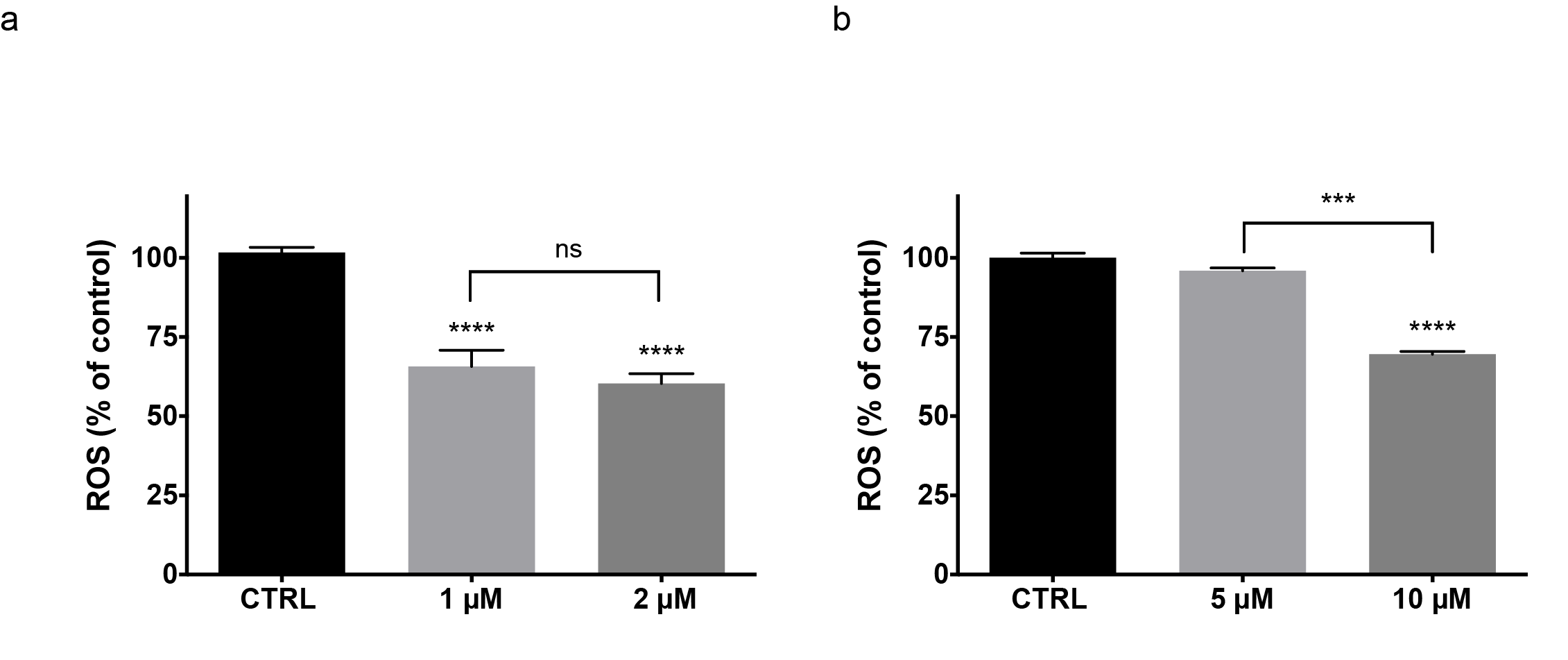


**Figure S1: Effect of the treatment with SF and EGCG on ROS intracellular level.**Cells were treated for 24 h with the indicated concentrations of SF (a) or EGCG (b). ROS level is evaluated by DCFH-DA fluorometric assay as reported in Materials and Methods. Each bar represents means ± SEM of 3 independent experiments. Data were analysed by one-way ANOVA followed by Bonferroni’s test., *** p< 0.001, **** p< 0.0001 with respect to control; ns, not significantly different
